# Evaluation of Suitable Reference Gene During the Development of Paired or Unpaired Female *Schistosoma japonicum* on the 18th and the 23rd Days Post Infection

**DOI:** 10.3390/pathogens14101066

**Published:** 2025-10-21

**Authors:** Suwen Wang, Liang Feng, Jun Sun

**Affiliations:** 1 Department of Life Sciences, Shihezi University, Shihezi 832002, China; wsw19880668@126.com (S.W.); fengliang515@163.com (L.F.); 2 Key Laboratory for Prevention and Control of Emerging Infectious Diseases and Public Health Security, The XPCC, School of Medicine, Shihezi University, Shihezi 832002, China; 3 NHC Key Laboratory of Prevention and Treatment of Central Asia High Incidence Diseases, School of Medicine, Shihezi University, Shihezi 832002, China; 4 Institute for Infectious Diseases and Vaccine Development, Tongji University School of Medicine, 500 Zhennan Road, Shanghai 200331, China

**Keywords:** *Schistosoma japonicum*, reference gene, *GAPDH*, *TUBA*

## Abstract

Background: Identifying optimal housekeeping genes is essential to accurately quantify gene expression dynamics across the 18th day (male and female begin to pair) and the 23rd day (female begin to sex mature) post infection of *Schistosoma japonicum*, because this process involves selecting suitable housekeeping genes to ensure the reliability and accuracy of all subsequent expression analyses, thereby improving the precision of biological interpretations. *Schistosoma japonicum* transcriptomics reveals marked stage-dependent variation in candidate reference genes, which directly challenges the long-standing hypothesis that commonly recommended reference genes remain stably expressed throughout the 18th day and the 23rd day post-infection developmental phases and therefore emphasizes the critical need for careful selection and rigorous validation in any specific experimental context. Methods: In this study, seven widely reported genes (*GAPDH*, *TUBA*, *ACTB*, *SOD1*, *TP*, *ND* and *PS*) of *Schistosoma japonicum* were systematically validated by combining Solexa high-throughput sequence analysis with targeted qPCR experiments to identify the most suitable reference genes on the 18th day and the 23rd day post infection of *Schistosoma japonicum*, and the expression stability of these seven candidate genes was then comprehensively evaluated using four complementary algorithms—the ΔCT method and the GeNorm V3.5, BestKeeper, and NormFinder software applications. Results: *GAPDH* displayed the most consistent expression profiles, whereas *TUBA* exhibited the least stability, particularly at the specific time points of 18 and 23 days post infection in both paired and unpaired female *Schistosoma japonicum*. Conclusions: The suitability of any housekeeping gene is strongly dependent on the study’s specific context and experimental conditions. Therefore, the conclusions drawn here are explicitly limited to the developmental window of 18 and 23 days post infection. Rigorous, stage-specific validation is indispensable before reliable quantitative gene expression analyses can be performed.

## 1. Introduction

Schistosomiasis, a primary parasitic disease, is only exceeded by malaria and tuberculosis in terms of the number of people it sickens and kills; approximately 200 million people are affected, who are spread across 76 tropical and subtropical nations [1,2,3]. The parasitic blood flukes have apparent differences between males and females and undergo a complicated development process [4,5,6]. The life cycle of the organism under scrutiny comprises seven distinctive stages, initiated by eggs, progressing to miracidia, followed by mother sporocysts, then daughter sporocysts, moving on to cercariae, subsequently juvenile schistosomula, and culminating in adult worms. The maturation and lifecycle of *Schistosoma japonicum* hinge on the pairing of male and female adults. During the developmental process of mice infected with schistosomiasis cercariae, the 18th and the 23rd days represent two key developmental stages [7]. Initially, on the 18th day, female and male worms begin to pair, although the female worm has not yet reached sexual maturity. Subsequently, by the 23rd day, the female worm has attained sexual maturity but has not yet started laying eggs. However, beyond the 23rd day, eggs will appear in the ovaries of the female worm. These eggs exhibit gene expression patterns that differ from those of the female worm’s own tissues. That is the primary reason why this study emphasizes the selection of reference genes specifically for these two key developmental stages. In order to explore the different gene expression levels of the female before and after the pairing, suitable reference genes are required.

It has been proposed that several reference genes should be considered [8,9,10]. However, some reference genes, such as glyceraldehyde-3-phosphate dehydrogenase (*GAPDH*), 18s, and *ACTB*, have been utilized extensively in relevant research [11,12,13,14,15,16]. Furthermore, a reasonable demonstration, which is related to transcription levels of reference genes, is not definite across various phases of development and under multiple experimental settings, as shown by many studies [17,18,19]. Undoubtedly, the inappropriate use of reference genes leads to false gene expression results [20,21]. Gene expression could alter or comply with developing periods. Using only one inappropriate housekeeping gene (HKG) has the potential to engender an inaccuracy [22]. Therefore, it is essential to select a suitable reference gene [23,24,25].

When selecting reference genes for quantification analysis, housekeeping genes (HKGs) are often chosen. These genes are constantly transcribed throughout the cell cycle and during different developmental periods [26]. However, little is known about which HKG can effectively reveal the characteristic gene expression level at a particular key developmental stage [27,28]. Properly selecting reference genes for specific experimental applications is essential for accurately analyzing a particular developmental stage for normalization [29]. For example, this study examined the impact of various recommended reference genes on the gene expression of four distinct developmental stages of female *Schistosoma japonicum* before and after infection.

## 2. Materials and Methods

### 2.1. Ethical Considerations

This investigation was conducted in strict compliance with the guidelines detailed in the Regulations for the Administration of Experimental Animals, as stipulated by the State Science and Technology Commission. The experimental protocol was reviewed and approved by the Ethics Committee of Tongji University School of Medicine. This study was conducted in accordance with the Declaration of Helsinki, and the animal study protocol was approved by the Ministry of Science and Technology’s “Regulations on Experimental Animal Administration” guidelines and the Tongji University School of Medicine’s Internal Review Board (Approval Number: TJLAC-014-017).

### 2.2. Unisexual and Paired Infections

We performed biological replicates and technical replicates. A total of 70 mice were divided into three batches, each infected with cercariae released from snails infected with a single sex. The 70 mice were used in three separate experiments: 20 mice were infected in the first experiment, 25 mice in the second experiment, and 25 mice in the third experiment, and the worms harvested from each mouse were processed separately.

*Oncomelania hupensis* were retrieved via the National Center for Parasitic Control and Research and the Chinese Center for Disease Control and Prevention. To generate mono-sex female worms, the snails were subjected to a solitary miracidium, which was produced in a laboratory setting from eggs obtained from the livers of rabbits or mice infected with Cercaria. To obtain both double-sex (cercariae released by the infected snails are both male and female, and the schistosomes from the infected mice include both male and female) and single-sex (cercariae released by the infected snails are all male or female, and the schistosomes from the infected mice are only male or female) female worms, it is necessary to release approximately 100–150 newly encysted cercariae from gastropods to percutaneously infect every mouse. The mice were euthanized humanely on 18 and 23 days subsequent to infection. The 18-day-old female worms from double-sex infections (18DSI), 18-day-old female worms from single-sex infections (18SSI), 23-day-old female worms from double-sex infections (23DSI), and 23-day-old female worms from single-sex infections (23SSI) were recovered and managed in accordance with the established methodology [24]. The worms were cleansed in a cold saline solution and examined under a microscope to ensure there were no unintended mixed-sex infections. Single-sex female worms were then isolated and stored at −80 °C for subsequent analysis. Female worms were isolated by washing in cold saline solution and carefully separating them from paired worms under a microscope. All samples were preserved at −80 °C for further processing.

### 2.3. RNA Extraction, Amplification, and Solexa Sequencing Analysis

RNA was extracted using the Trizol method. We began by flushing the schistosomes that were parasitizing within the mice from the hepatic and intestinal blood vessels into a culture dish, then washed them with physiological saline to remove impurities. Next, we transferred the female worm bodies one by one into RNase-free specialized EP tubes using a syringe, placing 20–25 female worms in each tube. We processed the worm bodies using RNase-free grinding rods that had been soaked overnight in 75% ethanol. Approximately 1000 female worms were used.

Total RNA (18SSI, 18DSI, 23SSI, 23DSI) was isolated by means of TRIzol reagent (Invitrogen Life Technologies, Carlsbad, CA, USA) in a manner consistent with the producer’s guidelines. The RNA concentration and purity metrics were determined through spectrophotometric assessment, utilizing absorbance readings at 260 nm to quantify the RNA levels and at 280 nm to appraise the purity grade, employing a NanoDrop ND1000 spectrophotometer (Thermo Fisher Scientific, Palo Alto, CA, USA) in conjunction with an Agilent 2100 Bioanalyzer (Agilent Technologies, a distinguished creator of sophisticated laboratory apparatuses for biomolecular scrutiny, located in Palo Alto, CA, USA).

The RNA specimens were stored at a temperature of −80 °C for long-term storage to maintain their integrity. The procedures for constructing the tag-seq library, handling the data, and determining the differentially expressed genes were also completed and performed according to the previous paper [17].

For cDNA synthesis, a 10 μL reaction mixture was prepared containing 2 μL of RNA (0.075 µg/µL), 2 μL of primer (1 µM), 4 μL of 5× PrimeScript Buffer, 1 μL of PrimeScript RT Enzyme Mix I, and 1 μL of RNase-Free Water. The reaction was performed under the following conditions: 16 °C for 30 min, 42 °C for 30 min, and 70 °C for 15 min, followed by 25 cycles. The synthesized cDNA was stored at −20 °C.

### 2.4. Gene Expression Validation via qPCR

For qPCR, a 10 μL reaction volume was used, and we performed technical triplicates for each sample. Oligonucleotide sequences targeting seven genetic loci were computationally generated using Primer Express 3.0 (Applied Biosystems, Foster City, CA, USA), with design criteria prioritizing amplicon lengths of 70–150 bp and theoretical Tm values of 60 ± 1 °C. Post-synthesis validation included melt curve analysis (95–60–95 °C), and the specificity of the seven target amplicons was confirmed through melt curve analysis (distinct single peaks) and electrophoretic validation on 1.5% agarose gels, which displayed sharp bands corresponding to predicted molecular weights. Primer efficacy for each pair was calculated via the Vector NTI software (Vector NTI 10.3) using fluorescence data obtained from quantitative PCR. All amplification reactions exhibited the optimal performance, with efficiencies exceeding 94%. All experimental specimens underwent systematic screening to detect primer–dimer formation, exogenous nucleic acid impurities, or mispriming by inspecting their dissociation curves. Table 1 enumerates every candidate gene’s complete designation, accession identifier, and functional classification. The reaction system and conditions for qPCR are detailed in Table 2 and Table 3, respectively. To ensure the reliability of the qPCR results, both a no-reverse transcription (No-RT) control and a non-template control (NTC) were included. The No-RT control was prepared by omitting the reverse transcriptase enzyme during the cDNA synthesis step. The NTC was prepared by replacing the cDNA template with RNase-free water. The qPCR was performed under standard cycling conditions, and the results were analyzed using the comparative Ct method to quantify gene expression levels.

### 2.5. Reaction Configuration

The 10 μL amplification system comprised the following:

Template: 2 μL cDNA (1:5 dilution).

Master Mix: 5 μL SYBR Green reagent (Agilent Technologies, Santa Clara, CA, USA).

Primers: 0.2 μL each (10 μM stock) (Table 1).

Volume Adjustment: 2.6 μL RNase-free H_2_O.

Non-Template Controls (NTCs):

In the reaction mixture, the cDNA template is omitted, and 2.6 μL RNase-free water is used for volume adjustment to maintain the total reaction volume of 10 μL.

No-RT Controls:

RNA samples that were not reverse transcribed.

### 2.6. PCR Cycling Conditions

Please refer to Table 2 for specific details.

### 2.7. Data Processing

We conducted qPCR experiments on seven candidate reference genes, collecting the corresponding Ct values to assess gene expression stability. Reaction efficiencies (90–110% acceptance range) were calculated via LinRegPCR (Version 2014.5) using cycle threshold (Ct) values from the exponential amplification phase. Cross-plate normalization was achieved by processing all replicates for individual genes on identical 96-well plates [30,31].

For data processing, we utilized Microsoft Excel 2019 software to perform the conversion of Ct values and the calculation of gene expression levels based on the raw Ct data. The average Ct values from three sets of three separate triplicate samples were used as the initial data. Then, the data were adjusted to the relative expression ratios using the Delta Cycle Threshold (ΔCT) method [12,21,32]. Prior to statistical analysis, we conducted normality tests on the Ct values using the Shapiro–Wilk test. This step ensured that the data met the prerequisite of normal distribution, thereby guaranteeing the validity of subsequent statistical analyses.

To compare gene expression stability across different developmental stages, we employed one-way Analysis of Variance (ANOVA) to evaluate differences between various sample groups. In cases where significant differences were identified, we further conducted Tukey’s Honest Significant Difference (HSD) test for multiple comparisons correction to ensure the accuracy of our results. In addition, we present the expression levels of seven genes across different developmental stages using *GAPDH* and *TUBA* as reference genes, with error bars representing the standard error (SE). The SE was calculated by averaging the standard deviations from three technical replicates per group, which helps reflect the variability in the experimental data.

Gene expression stability analysis and evaluation were performed by the geNorm v3.5 software as previously described [20,26,33]. The program is able to calculate an expression stability value (M) for each gene. Genes with higher M values are considered to be expressed less stably [34]. A multivariate analysis platform (https://blooge.cn/RefFinder/?type=reference, accessed on 1 January 2025) integrating ΔCT quantification and the BestKeeper, NormFinder, and geNorm algorithms [26,35,36,37,38] generated stability indices for reference genes. Algorithmic prioritization identified benchmark genetic candidates, with subsequent mRNA quantification performed via Bioanalytical Suite v1.4 (Applied Biosystems).

We comprehensively assessed the expression stability of these genes using a variety of algorithms (the ΔCT method, GeNorm, BestKeeper, and NormFinder), ensuring the reliability of our analytical results. Through these methods, we were able to visually compare the expression stability of these candidate reference genes during different developmental stages of the parasites, thereby providing support for subsequent gene expression analysis.

## 3. Results

**Figure 1:**
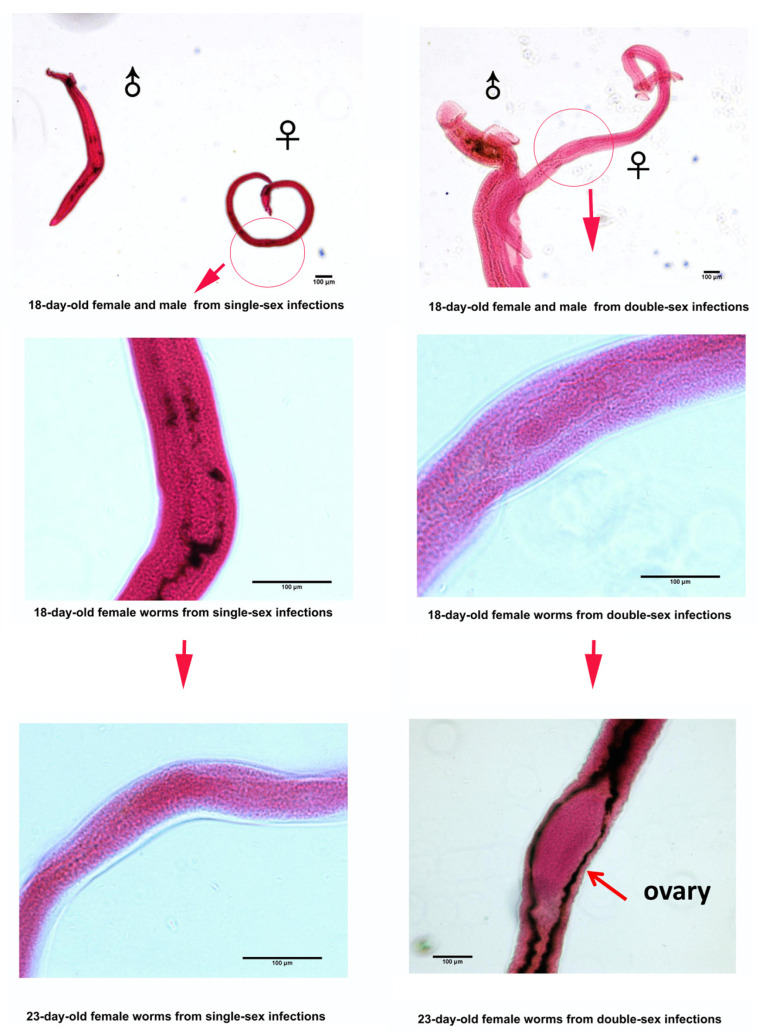
The four stages shown are female of *Schistosoma japonicum* stained using Carmine Red. On the 18th day and the 23rd day from a single-sex female, no ovaries are present; whereas, on the 23rd day from a double-sex female, an ovary appears, preparing for the subsequent production of eggs. The short arrows in the four subfigures above indicate that the female worms are continuing to develop. The two circles at the top represent the location of the ovaries, but they are not yet mature. The long arrow in the lower right corner indicates the mature ovary of the female worm.

The female and male of *Schistosoma japonicum* begin to pair on the 18th day after infecting the mouse. Pairing promotes the sexual maturation of the female, which reaches sexual maturity on the 23rd day after infection, with the ovary fully developed but without any eggs. The results are shown in Figure 1.

**Figure 2:**
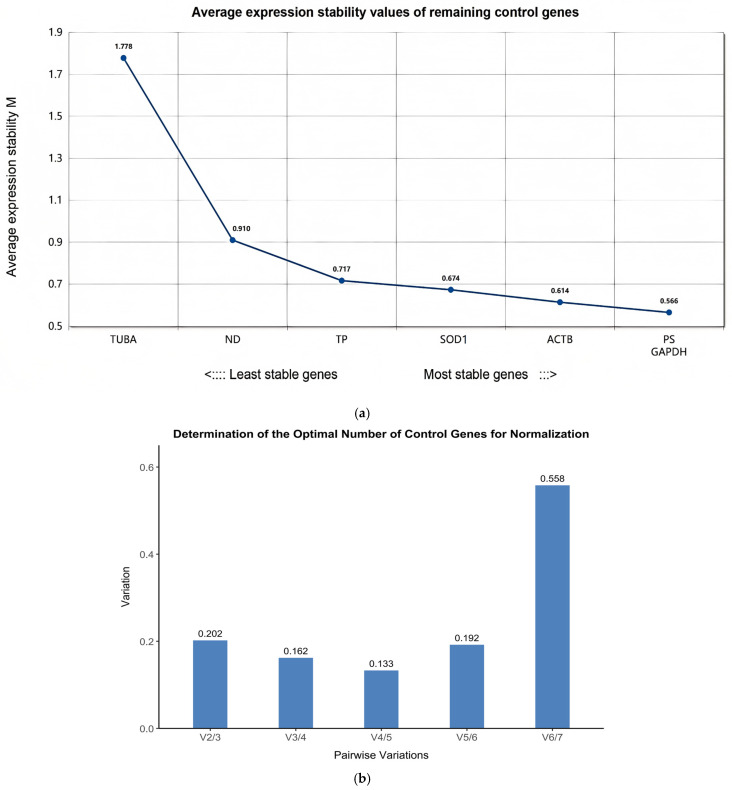
(**a**) The expression stability of seven candidate reference genes across four specific developmental stages of *Schistosoma japonicum* female worms (18SSI, 18DSI, 23SSI, 23DSI), as analyzed by the geNorm software. The horizontal axis represents the ranking of gene stability, from least stable (left) to most stable (right). The vertical axis indicates the average expression stability value (M value), with the recommended criterion being that genes with an M value less than 1.5 are acceptable as reference genes. Lower M values denote more stable gene expression. The figure clearly labels the M value for each gene, with *GAPDH* and *PS* showing the lowest M values, indicating that they have the highest expression stability across the studied developmental stages, and their M values are both below the recommended threshold of 1.5, thus making them suitable reference genes. (**b**) The pairwise variation (V) analysis conducted by geNorm software to determine the minimum number of reference genes needed for gene expression analysis across the four developmental stages of *Schistosoma japonicum* females. The horizontal axis represents the pairwise variations between different gene pairs (V2/3, V3/4, V4/5, V5/6, V6/7), and the vertical axis represents the V values. The cutoff value is 0.15, which is the threshold recommended by geNorm analysis for determining the number of reference genes required. According to the data shown in the graph, V2/3 = 0.202, V3/4 = 0.162, V4/5 = 0.133, V5/6 = 0.192, and V6/7 = 0.558. The V4/5 value is the first pairwise variation value that falls below the 0.15 threshold, theoretically indicating that four reference genes are needed to ensure robust normalization. However, considering experimental resources and operational convenience, this study selected the most stable gene and the most unstable gene (*GAPDH* and *TUBA*) for normalization. Nonetheless, we acknowledge that using four genes might provide more reliable normalization results, and we recommend considering this in future research.

The expression robustness of seven distinct genes was meticulously appraised through the application of geNorm software, specifically version 3.5, which calculates M values as indicators. According to geNorm’s guidelines, an M value of 1.5 is typically used as a cutoff for identifying stable reference genes [39]. In the context of this study, six out of the seven selected reference genes exhibited M values below the threshold of 1.5, indicating their relative stability. The exception was the gene *TUBA*, which had an M value above this cutoff. Our analysis revealed that *GAPDH* and *PS* had the smallest M values, suggesting they were the most stable, whereas *TUBA* had the largest M values, indicating that its expression was unstable during the parasite’s developmental stages (Figure 2a).

The cutoff value of 0.15 indicates that the inclusion of additional reference genes does not significantly improve normalization accuracy. V2/3 = 0.202, V3/4 = 0.162, V4/5 = 0.133, V5/6 = 0.192, and V6/7 = 0.558. The geNorm results showed that V4/5 was approximately 0.14, marking the first paired variation value below the 0.15 threshold (Figure 2b). The lowest V4/5 value below 0.15 suggests that four housekeeping genes (*GAPDH*, *PS*, *ACTB*, *SOD1*) should be selected for reliable normalization.

**Figure 3:**
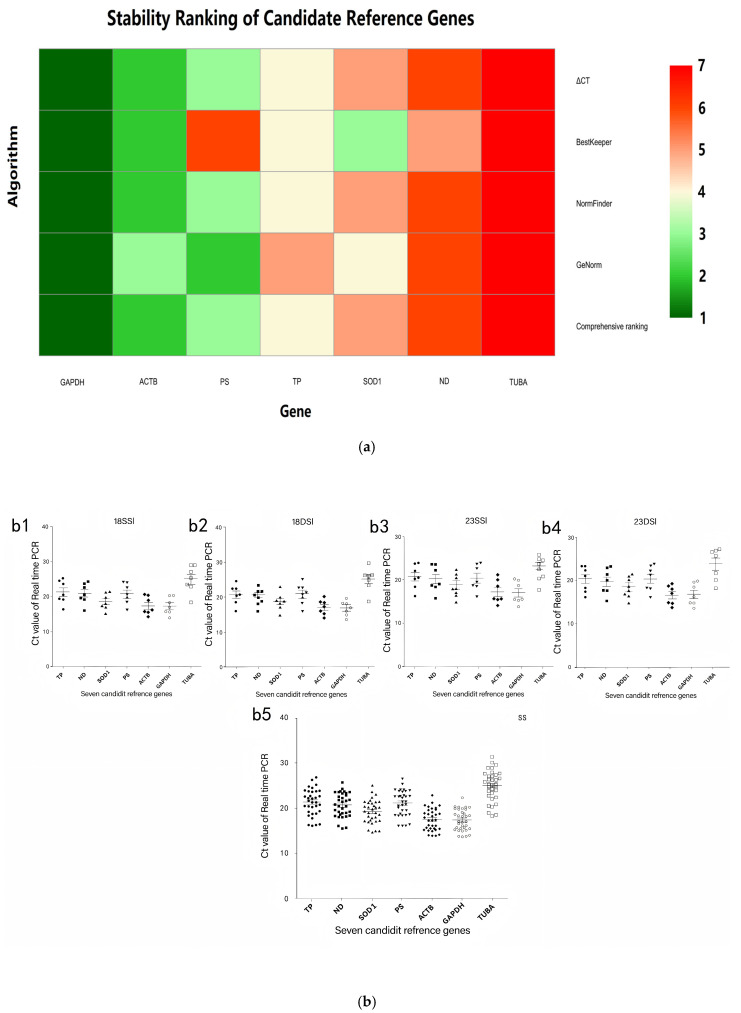
(**a**) The heatmap illustrates the expression stability of seven candidate reference genes across four developmental stages of *Schistosoma japonicum* females (18SSI, 18DSI, 23SSI, 23DSI). The color gradient in the heatmap ranges from green (indicating high stability) to red (indicating low stability), reflecting the stability of gene expression. The numbers 1 to 7 next to the color bar represent the stability ranking of the genes, with smaller numbers indicating more stable gene expression. It can be observed from the figure that *GAPDH* exhibits higher stability across all developmental stages, while *TUBA* shows relatively lower stability, suggesting that *GAPDH* may be more reliable choices for reference genes; (**b**) real-time PCR Ct values of seven reference genes in *Schistosoma japonicum* female worms across four distinct developmental stages. Each subplot (**b1**–**b4**) corresponds to a specific stage, with the scatter plots indicating the Ct values of each gene across various samples. Each dot represents a technical replicate, and the error bars denote the standard deviation of the samples. The Ct value is the cycle number at which the fluorescence signal in real-time PCR reaches a preset threshold; a lower Ct value typically signifies a higher level of gene expression and better amplification efficiency. Across all developmental stages, *GAPDH* generally exhibits lower Ct values, suggesting it has a higher initial copy number in the samples and good amplification efficiency, making it a good choice for a reference gene. In contrast, *TUBA* usually has higher Ct values, which may indicate a lower initial copy number in the samples or lower amplification efficiency compared to *GAPDH*, and thus its suitability may need to be considered more cautiously when selecting a reference gene. Subplot (**b5**) compiles data from all developmental stages, further comparing the distribution of Ct values among the genes, emphasizing the importance of considering the Ct value distribution when choosing reference genes.

In addition, genes are arranged from the most stable (top) to the least stable (bottom). *GAPDH* was the most stable genes across all four analytical platforms. *ACTB*, *PS*, and *SOD1* exhibit intermediate stability, whereas *TUBA* was ranked as the least stable. The convergent evidence from multiple algorithms underscores the reliability of selecting *GAPDH* and *TUBA* as the reference gene pair for qPCR normalization in gene expression studies targeting the 18–23 days post-infection developmental window of female *Schistosoma japonicum* (Figure 3a).

However, to gain a more thorough understanding of the genes’ stability, an integrated analysis was conducted using a combination of tools: geNorm, NormFinder, BestKeeper, and the ΔCT method. Based on this analysis, the genes were ordered from most to least stable as follows: *GAPDH* > *ACTB* > *PS*> *TP* > *SOD1* > *ND* > *TUBA* (Table 4). Furthermore, the expression patterns of these seven reference genes were investigated across four developmental stages of *Schistosoma japonicum* (18SSI, 18DSI, 23SSI, and 23DSI), with the findings presented in Figure 3b.

Due to the limited availability of female schistosome material and the high cost of reagents, we initially only validated the most (*GAPDH*) and least (*TUBA*) stable reference genes across the four developmental stages. Once the reliability of *GAPDH* has been confirmed, subsequent large-scale experiments will include *PS*, *ACTB* and *ND* to meet the four-gene normalization standard recommended by geNorm.

**Figure 4:**
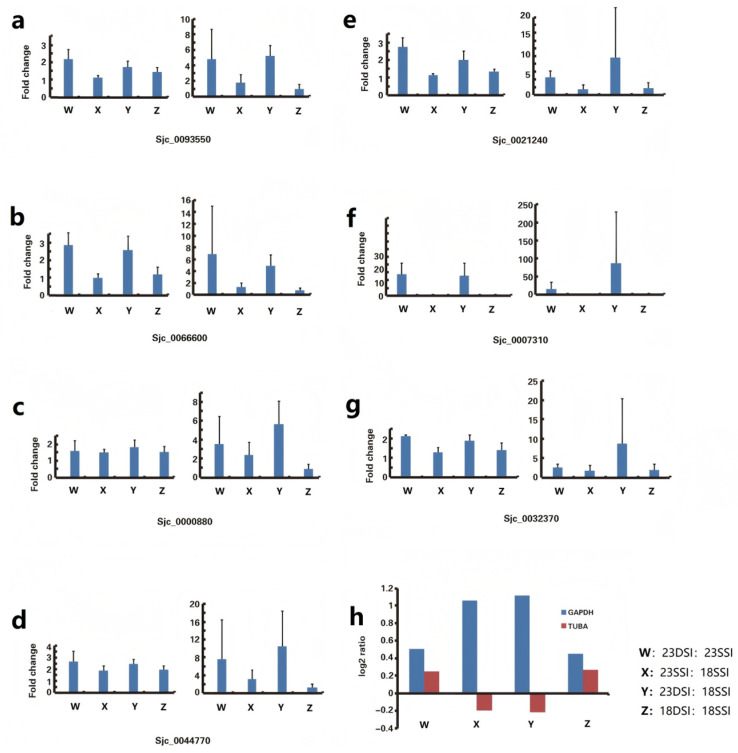
(**a**–**g**) Bar graphs represent the relative expression levels of seven target genes (*Sjc_0093550*, *Sjc_0066600*, *Sjc_0000880*, *Sjc_0044770*, *Sjc_0021240*, *Sjc_0007310*, *Sjc_0032370*) normalized to *GAPDH* and *TUBA* across four developmental stages of *Schistosoma japonicum* (DSI23, SSI23, DSI18, SSI18). Error bars indicate the standard error of the mean. The data reveal significant variability in expression levels when normalized to *TUBA* compared to *GAPDH*, suggesting the latter’s superior stability as a reference gene. (**h**) Comparative Solexa sequencing analysis of *GAPDH* and *TUBA* expression levels across the same developmental stages. The log ratio highlights the differential expression patterns, with *GAPDH* showing more consistent expression across samples, reinforcing its suitability as a reliable reference gene in qPCR studies.

To explore the dependability of outcomes, we used qPCR to amplify seven other genes, namely, *Sjc_0093550*, *Sjc_0066600*, *Sjc_0000880*, *Sjc_0044770*, *Sjc_0021240*, *Sjc_0007310*, and *Sjc_0032370* (Table S1), with *GAPDH* and *TUBA* as reference genes in the four samples of *Schistosoma japonicum*. The CT values showed that the *TUBA* amplification results had the highest volatility, and the *GAPDH* results had the smallest (Figure 4a–g) (Table S1). Solexa analysis showed different *GAPDH* and *TUBA* expression levels in DSI23, SSI23, SSI18, and SSI18, uncovering the underlying cause for the discrepancies in results when these genes were utilized as internal reference genes (Figure 4h).

**Figure 5:**
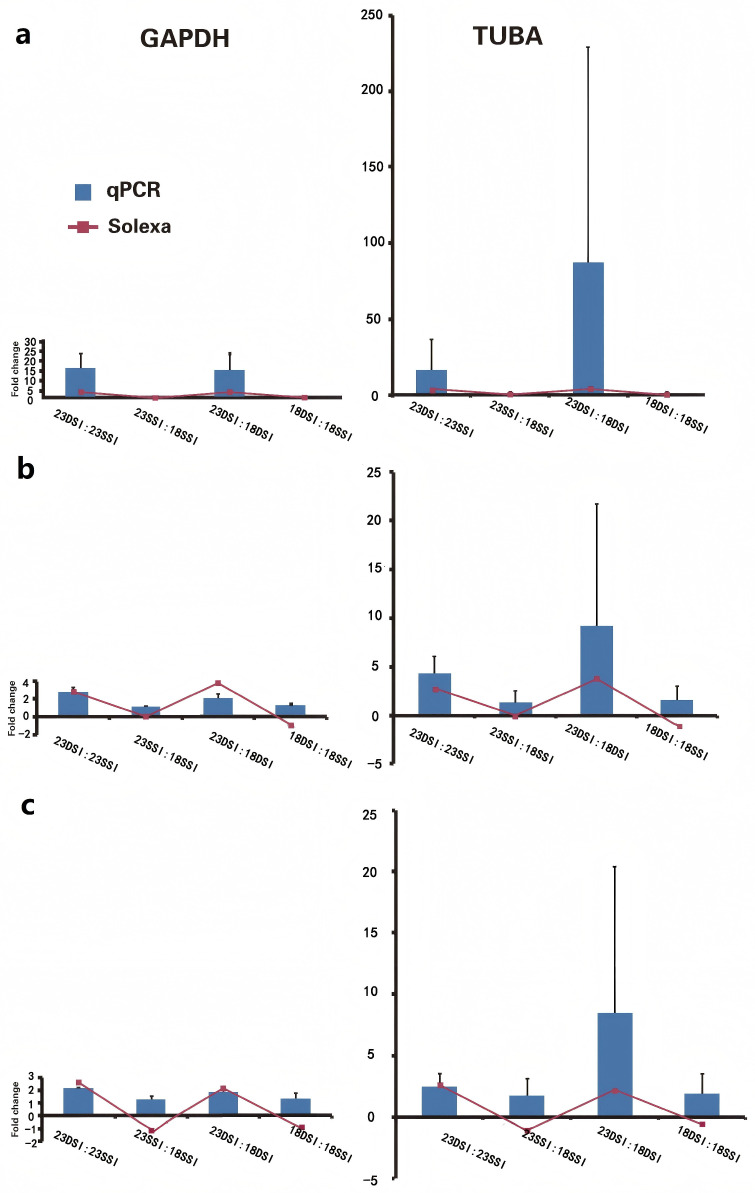
(**a**) Ferritin-1 Heavy Chain: The bar graph illustrates the expression levels of the ferritin-1 heavy chain gene normalized to *GAPDH*, as determined by qPCR (blue bars). The red line with circles represents the expression levels obtained from Solexa sequencing. Error bars represent the standard deviation, showing a correlation in trends between qPCR and Solexa, with *GAPDH* as a stable normalizer. (**b**) Major Egg Antigen (*p40*): This graph compares the qPCR results (blue bars) with Solexa sequencing data (red line with circles) for the major egg antigen gene, normalized to *GAPDH*. The close alignment of the data points suggests that *GAPDH* provides a reliable normalization, despite some variability. (**c**) Thioredoxin Peroxidase: The expression levels of the thioredoxin peroxidase gene, as measured by qPCR and normalized to *GAPDH*, are shown in blue. The Solexa sequencing results are indicated by the red line with circles. The consistency between the two methods, especially with *GAPDH* normalization, indicates its suitability as a reference gene.

The transcriptional activities of three specific genes—thioredoxin peroxidase, *p40* (major egg antigen), and the heavy chain of ferritin-1—were assessed using Solexa sequencing and quantitative PCR (qPCR) techniques. *GAPDH* and *TUBA* acted as the reference genes. The results showed that both qPCR results were similar to those of Solexa in the trend, but the results of *TUBA* exhibited more volatility (Figure 5). Bars represent the mean ± SD.

## 4. Discussion

The expression pattern of constitutively expressed genes in tissues and cellular environments tends to be highly stable. These genes are routinely utilized as a standard or benchmark when assessing alterations in gene expression profiles. Their primary purpose is to normalize variations arising from differences in sample input and experimental inconsistencies during the sample preparation phase, thus ensuring the fidelity of the experimental data. By quantifying these internal standards in each specimen, it becomes feasible to compensate for loading variations, thereby bolstering the credibility of semi-quantitative assessments. Nevertheless, it should be emphasized that certain widely utilized reference genes may not consistently function as optimal selections across every experimental scenario [40,41]. Thus, standardized references should be established in quantitative studies on transcriptional profiling during essential development stages, for example, the 18th and the 23rd days.

This study explored the four reference genes’ expression analysis tools (geNorm, NormFinder, BestKeeper, ΔCT) to analyze *TUBA*, *GAPDH*, *ACTB*, *PSMD4*, *NDUFV2*, *SOD1*, and *TPC2L* of four development stages of *Schistosoma japonicum*, to find an appropriate reference gene for our further study. Seven genes related to growth, development, and metabolism were chosen from the gene of *Schistosoma japonicum* for further analysis [42,43,44].

While our initial analysis highlighted *GAPDH* as a highly stable reference gene, particularly during the 18th and the 23rd days after infection, it is important to note that the geNorm analysis recommends using multiple reference genes for more robust normalization when V4/5 < 0.15. Therefore, although *GAPDH* is a strong candidate, when experimental materials and conditions permit, the use of additional reference genes, such as *PS*, *ACTB*, and *SOD1*, is recommended for comprehensive and reliable gene expression studies. We acknowledge that geNorm formally recommends the use of four reference genes when V4/5 < 0.15 (Figure 2b). However, for this proof-of-concept study, we restricted validation to the most stable (*GAPDH*) and the least stable (*TUBA*) candidates. First, the heat map in Figure 3a demonstrates that *GAPDH* consistently ranked first across all algorithms, whereas *TUBA* ranked last, providing an unambiguous visual contrast. Second, female schistosome material is scarce, and reagents such as SYBR Green and reverse transcriptase are costly; limiting the panel to two genes therefore maximized the biological material available for the experimental questions of primary interest. This two-gene strategy has been adopted previously in similar resource-limited settings [39,45]. Finally, the current validation is intended only as an initial benchmark; once *GAPDH*’s reliability is confirmed, *PS*, *ACTB*, and *SOD1* will be added in subsequent large-scale experiments to reach the full four-gene normalization system recommended by geNorm. However, due to experimental resource constraints, we only selected the top-ranked reference gene (*GAPDH*) for validation. Although *GAPDH* demonstrated high stability in our study, using a single reference gene may limit the reliability of the results. Therefore, we recommend using multiple reference genes when resources permit to enhance the reliability of the findings.

Our results differ from previous studies’ results [46,47]. In our investigation, the genes demonstrating the utmost stability were discerned to be *GAPDH* and *PS*, which manifested consistent expression patterns across the studied samples, and the most unstable ones were *ND* and *TUBA*. Previous studies covered every developmental stage, and the reference genes *PS* and *ND* were identified as those displaying the highest expression stability [46]. In contrast, the genes characterized by the most pronounced expression instability were *ACTB* and *TUBA*. These studies did not use *GAPDH*, even though it is the best reference gene when analyzing the gene expression at specific developmental stages, namely, 18SSI, 18DSI, 23SSI, and 23DSI. The difference between our results and the findings of previous studies was due to the choice of development stages [48,49].

To examine how the choice of reference genes affects the findings from qPCR analyses, an analysis was implemented to evaluate the relative expression patterns of three specific genes, namely, *ACE06819.1*, *CAX77721.1*, and *CAX72575.1*, during *Schistosoma japonicum*’s different developmental stages, using *GAPDH* and *ACTB* for analysis [50]. The results of the TUBA analysis were larger—even two or three times larger—than those of the *GAPDH* analysis. This revelation underscores that the disparate choice of reference genes profoundly influences the reproducibility and accuracy of experimental outcomes, thereby impacting the overall credibility of the findings. Though the same cDNA sample may be used in the experiment, different gene expression analysis results will be obtained, and the experimental time will be prolonged due to the different reference genes selected. Moreover, this study also proved that different or even opposite results will be obtained when different reference genes are used, leading to inaccurate results [51].

In addition, *GAPDH* and *TUBA* served as reference genes, which show gene expression patterns similar to those from Solexa sequencing. However, the results from the *GAPDH* group were more stable than those from the *TUBA* group. This suggests that although *TUBA* is a widely used reference gene, it is unsuitable for analyzing the current development stage. Discovering a reference gene well-suited for evaluating gene expression throughout all stages of development is an extremely unlikely endeavor, especially for *Schistosoma japonicum* with large morphological changes in its life history. This problem may also exist in other organisms with a complex life cycle.

Moreover, this study underscores that a single reference gene is not sufficient for every developmental stage. It is essential to experimentally verify the appropriate reference genes for each specific developmental stage, as this is crucial for the accuracy of quantitative gene analysis. In other words, when studying multiple developmental stages, selecting just one reference gene may not be adequate. Instead, multiple different reference genes may need to be chosen to jointly analyze the relative expression of genes. In our study, *GAPDH* was found to be relatively stable in the gene quantification analysis on days 18 and 23 post infection with *Schistosoma japonicum*. The reference gene should be selected at a relatively stable development stage with similar morphologies rather than at a stage with entirely different morphologies and physiological conditions, such as between eggs and adult worms, eggs and cercariae, or adult worms and miracidia.

## Figures and Tables

**Table 1:** Descriptions of candidate reference genes and primers for qRT-PCR.

Gene Symbol	Gene Production Name	Function	GenBank Accession Number	Primer Sequence (5′–3′) Forward/Reverse	Amplicon Length (bp)	Amplification Efficiency (%)	R^2^
*ACTB*	Beta-actin	Cytoskeletal structural protein	FN315418	TGGTAGCACAATGTATCCTGG/GCCTCAGGACAACGGAACC	115	95	0.98
*GAPDH*	Glyceraldehyde-3-phosphate dehydrogenase	Oxidoreductase in glycolysis and gluconeogenesis	FN324551	ATGGAACAAGGATGGTGCTGAG/CAACAAACATGGGTGCGTCT	143	95	0.96
*NDUFV2*	NADH dehydrogenase (ubiquinone) flavoprotein 2	Electron transport in respiratory chain	FN320220	CGAGGACCTAACAGCAGAGG/TCCGAACGAACTTTGAATCC	174	95	0.99
*PSMD4*	26S proteasome non-ATPase regulatory subunit 4	Binds and presumably selects ubiquitin conjugates for destruction	FN320595	CCTCACCAACAATTTCCACATCT/GATCACTTATAGCCTTGCGAACAT	129	95	0.97
*TPC2L*	Trafficking protein particle complex subunit 2-like protein	May play a role in vesicular transport from endoplasmic reticulum to Golgi apparatus	FN319821	CTCAAGCAGCCCAGTTCAGT/AATCGGTGTGCCAGGTTTAT	137	95	0.95
*TUBA*	Tubulin alpha	The major constituent of microtubules	AY815746	TGGAACATTCTGATTGTGCCT/CGATGCCGTAATAGAACTAACA	140	95	0.97
*SOD1*	Superoxide dismutase 1	Destroys free superoxide radicals	FN315362.1	CTGATGACGGAAAGGGAG/CTATGACACCACAAGCTACA	462	95	0.98

**Table 2:** Amplifications conditions on the 7300 Real-Time PCR System (Applied Biosystems).

Phase	Temperature (°C)	Duration	Function
1	95 °C	30 sec	Enzyme activation and Denaturation
2	95 °C → 60 °C	40 cycles	PCR Cycles
3	60 °C → 95 °C	Melting Curve	Specificity verification

Technical triplicates were processed per sample to minimize intra-assay variability.

**Table 3:** Full thermocycling details of Real-Time PCR System.

Step	Temperature (°C)	Time (s)	Number of Cycles
Pre-denaturation	95	30	1
Denaturation	95	5	40
Annealing	60	34	40
Extension	60	34	40
Hold	−20	Hold	-

**Table 4:** Ranking of candidate reference genes according to their stability value using ΔCT, BestKeeper, NormFinder, and geNorm integrated analysis.

Method	Ranking Order						
	← Most Stable Genes				Least Stable Genes →		
	1	2	3	4	5	6	7
ΔCT	*GAPDH*	*ACTB*	*PS*	*TP*	*SOD1*	*ND*	*TUBA*
BestKeeper	*GAPDH*	*ACTB*	*SOD1*	*TP*	*ND*	*PS*	*TUBA*
NormFinder	*GAPDH*	*ACTB*	*PS*	*TP*	*SOD1*	*ND*	*TUBA*
GeNorm	*GAPDH/PS*	*-*	*ACTB*	*SOD1*	*TP*	*ND*	*TUBA*
Comprehensive ranking	*GAPDH*	*ACTB*	*PS*	*TP*	*SOD1*	*ND*	*TUBA*

The arrows pointing left indicate that the genes become more stable the further to the left they are; the arrows pointing right indicate that the genes become less stable the further to the right they are.

## Data Availability

All raw data and code are available upon request.

## Supplementary Materials

The following supporting information can be downloaded at https://www.mdpi.com/article/10.3390/pathogens14101066/s1, Table S1: Comparison of expression level of 7 genes using Solexa among 18SSI, 18DSI, 23SSI, and 23DSI of *Schistosoma japonicum*.

## Abbreviations

The following abbreviations are used in this manuscript:

*ACTB*	Beta-actin
*GAPDH*	Glyceraldehyde-3-phosphate dehydrogenase
*NDUFV2*	NADH dehydrogenase (ubiquinone) flavoprotein 2
*PSMD4*	26S proteasome non-ATPase regulatory subunit 4
*TPC2L*	Trafficking protein particle complex subunit 2-like protein
*TUBA*	Tubulin alpha
*SOD1*	Superoxide dismutase 1
18SSI	18-day-old female worms from single-sex infections
18DSI	18-day-old female worms from double-sex infections
23SSI	23-day-old female worms from single-sex infections
23DSI	23-day-old female worms from double-sex infections
qPCR	Quantitative polymerase chain reaction
